# Light Scattering by a Subwavelength Plasmonic Array: Anisotropic Model

**DOI:** 10.3390/s22020449

**Published:** 2022-01-07

**Authors:** Anton Nemykin, Leonid Frumin, David Shapiro

**Affiliations:** Institute of Automation and Electrometry, Russian Academy of Sciences, Siberian Branch, 1 Koptjug Ave, 630090 Novosibirsk, Russia; nemykin@iae.nsk.su (A.N.); lfrumin@iae.nsk.su (L.F.)

**Keywords:** plasmonic array, subwavelength grating, biosensors, anisotropic permittivity, hyperbolic metamaterials

## Abstract

We calculate the light transmission by a subwavelength plasmonic array using the boundary element method for parallel cylinders with different cross-sections: circular or elliptic with axis ratio 4:1. We demonstrate that plasmonic resonance is sharper for the case of horizontal ellipses. This structure is susceptible to refractive index variations in the media since the high derivatives of reflection and transmission coefficients are near the angle of total internal reflection. To obtain an approximate analytical expression, we used the model of a metallic layer. We explore the “sandwich” structure with an anisotropic film between two dielectrics and demonstrate its quantitative agreement with numerical results.

## 1. Introduction

Plasmons are surface waves of conduction electrons inside the metallic film with a dielectric border. The dispersion relation for plasmons differs from the bulk plasma due to an interaction with the dielectric medium. An electric field decays when one penetrates deeper into the metal and dielectric areas. At plasmon resonance, there is a notable increase in electric field intensity [1]. There is important progress in the light scattering study of small particles with plasmon excitation [2], their dimers [3], or other plasmonic nanostructures [4].

Applications of surface plasmons are diverse: from optical biosensors [5,6,7] to the acceleration of relativistic electrons [8] and space jet engines [9]. A number of applications are based on the symmetry broken structures: the “hybridisation” of plasmons, i.e., the interaction of elementary surface waves supported by nanostructures [10,11,12].

Let us consider the lower dielectric half-space as the substrate with a planar periodic structure on top. The refractive index of the upper half-space strongly affects the angular and wavelength spectral characteristics of the scattering layer due to crucial changes in layer effective permittivity. These resonance responses underlie plasmon biosensors. Scattering on a layer is also essential for research in silicon photonics, such as the study, development, and manufacture of optical microcircuits in which photons propagate instead of electrons [13]. All-optical circuits can significantly enhance the density of communication channels and lead to considerable energy savings.

Recently, the study of the plasmon–enhanced local field in the array of parallel circular metallic cylinders was proposed to improve sensitivity to refractive index variations [14] regarding rapid changes in the Fresnel coefficients near the angle of total internal reflection (TIR). We had analyzed the grid of parallel metallic cylinders between two dielectrics; see [15] and references therein. The idea of the boundary element method (BEM) is to reduce the Maxwell equations to boundary integral ones applying the Green theorem. In the case of a grid with parallel cylinders, the Floquet theorem reduces the problem of one cylinder within one elementary cell. To obtain analytical formulas, we exploited simplified models. The sequence of nanowires can be approximately replaced by a thin layer with averaged permittivity [16]. The model offered approximate angular dependence but never yielded quantitative agreement. In the present paper, we treat more general models of a layer with an anisotropic dielectric tensor. Cylinders of the elliptical cross-section with different orientations are compared with the model, demonstrating its advantage.

## 2. BEM Calculation

We carried out numerical modelling with the BEM [15] for cylinders near the interface between the upper free half-space ($\varepsilon_{2}=1$) and glass lower half-space $\varepsilon_{1}=2.25$. The original codes were developed on the basis of the effective Green function of dielectric half-space [15] that allows for us to avoid integration over the infinite interface. We analyzed a grating with period *d* consisting of gold ($\varepsilon_{3}=-23.6+i1.27$ at λ=0.7749μm). We chose the wavelength according to two conditions: (i) data were present in the handbook [17] by Palic; (ii) the absolute value of the real part to imaginary part ratio of dielectric constant should be maximal. The latter is necessary to provide a large Q-factor.

**Figure 1:**
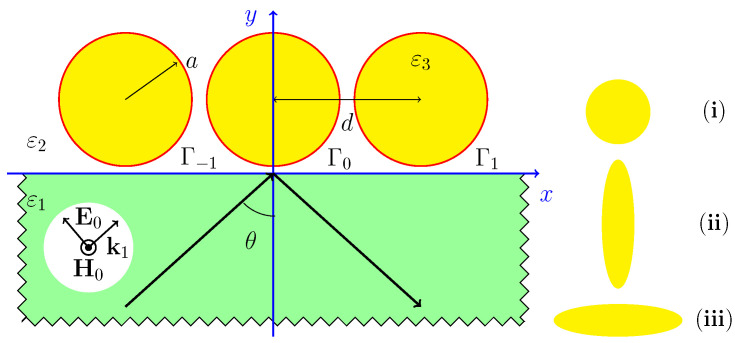
Periodic set of circular cylinders near the interface between free space and glass. Inset describes the considered cross-sectional shapes: (**i**) circle, (**ii**) vertical ellipse, (**iii**) horizontal.

We considered three shapes of cylinder cross-sections: circle of radius a=0.05μm and ellipses with axial ratio 1:4 and 4:1, as shown in the inset in Figure 1. The cross-sectional area was fixed, and the gap dimension was 0.01μm for all the cases; the period was different. This was d=0.11μm for circular cylinders, 0.06μm for vertical ellipse, and 0.21μm for the horizontal. The distance between their centers and the glass half-space was a fixed 0.11μm for all cases. The incidence field was *p*-wave near angle $\theta =\theta_{0}=41.81^{\circ }$, where $\theta_{0}$ is the TIR angle. In calculations, the grid sampling was nonuniform near $\theta_{0}$ by a power law with exponents 3/2.

Formulas for the extinction and scattering are

(1) $$C_{ext}=-\frac{1}{I_{0}}\int \left(S_{ext}\cdot e_{r}\right)\;dA,\;C_{s}=\frac{1}{I_{0}}\int \left(S_{s}\cdot e_{r}\right)\;dA,$$

where $S_{s}=\left[E_{s}\times H_{s}^{*}\right]$, $S_{ext}=\left[E_{0}\times H_{s}^{*}+E_{s}\times H_{0}^{*}\right]$ are the Pointing vectors of scattered radiation and the energy flux of interaction between scattered electric and magnetic fields $E_{s},H_{s}$ and incident fields $E_{0},H_{0}$, responsible for the extinction, $e_{r}$ denotes the radial unit vector. We take the integral over surface *A* of a cylinder. The absorption coefficient was calculated as the difference $C_{a}=C_{ext}-C_{s}$ [18,19]. FEF and absorption had close qualitative behaviour. The transmission coefficient was calculated in the far field domain, neglecting all evanescent modes.

**Figure 2:**
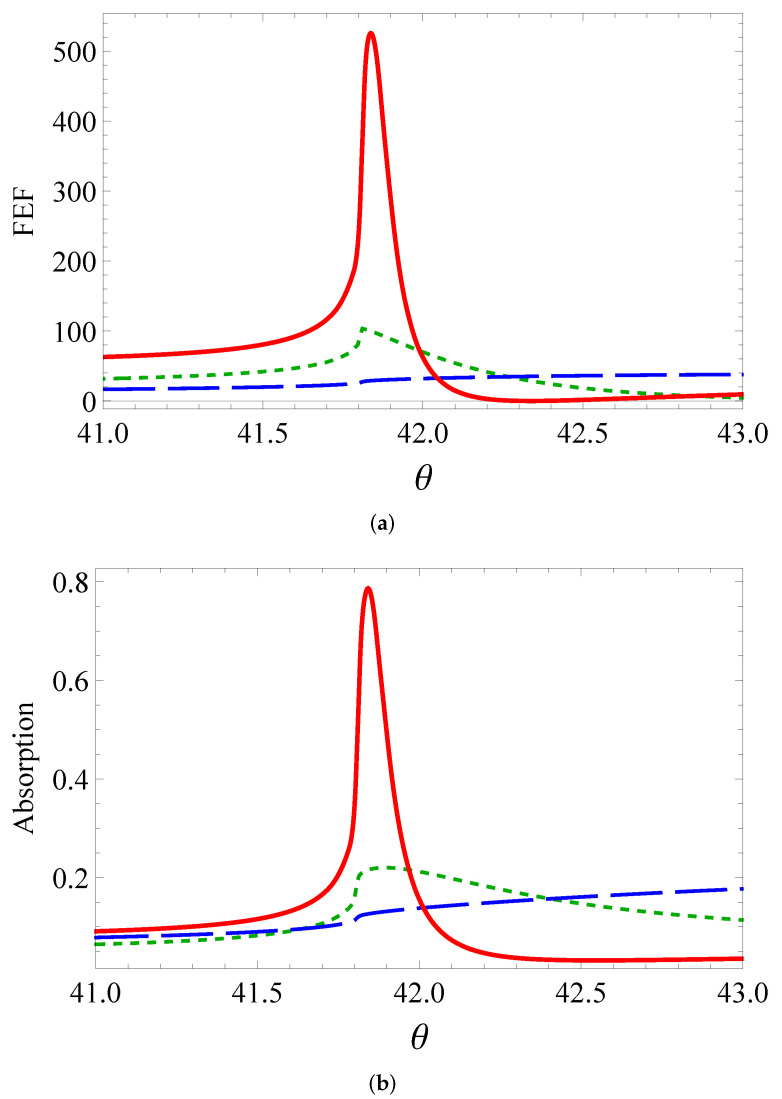

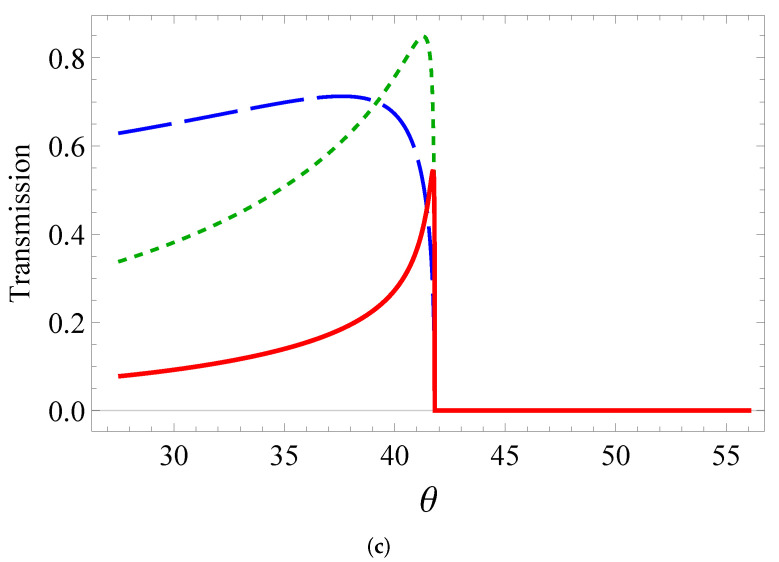
(**a**) Field enhancement factor, (**b**) absorption, and (**c**) transmission as a function of incidence angle θ (degrees): circles (short dashes), vertical ellipses (long dashes), horizontal ellipses (solid line).

Figure 2 shows a comparison of the angular dependencies near the TIR angle of the (a) field enhancement factor (FEF), (b) absorption, and (c) transmission coefficients for three versions of cross-sections. Figure 2a demonstrates the $\mathrm{FEF}=|E/E_{0}{|}^{2}$ in the middle between neighbor cylinders as a function of the incidence angle. Here, ***E*** is the electric field in the middle of the gap, and $E_{0}$ is the incident field. Plasmon resonance induces an exceptionally sharp peak for the horizontal ellipses. The highest peak was observed since the horizontal ellipse had a minimal radius of curvature in the gap. The curvature radius near the slit was $6\times 10^{-3},5\times 10^{-2},4\times 10^{-1}\;\mu$m, for the horizontal ellipse, circle, and vertical ellipse, respectively. That is why the case of the horizontal ellipse yielded a sharp peak in FEF and absorption, whereas the peak for the vertical ellipse practically vanished. The circle was intermediate between the limiting cases.

As previously mentioned [14], the resonance of the slit grating is excited by the refracted into the upper half-space (Fresnel) field. There was only an evanescent wave when θ exceeded the TIR angle. As a result, the Fresnel field achieved its maximum near the TIR angle; its *x*-component vanished. The absolute maximum of the intensity is created only by the *y* component of the Fresnel field; *x* component $E_{x}$, and not $E_{y}$, experiences resonance in the gaps. A natural question arises: how is this possible if the Fresnel *x*-component of the field vanishes? The field scattered on the cylinders had a significant *x* component. Since the cylinders were not limiting the subwavelength (kd=0.9,0.5,1.7 for cases (i)–(iii)), then the scattering differed from that in the dipole case. The phase difference between the neighbour wires originated the additional contribution to the *x* component. The gaps between the cylinders were a subwavelength: kΔ<1, where Δ=0.01μm.

Absorption in Figure 2b correlated well with FEF in Figure 2a. The most intensive electric field and accordingly the largest damping were precisely achieved in plasmon resonance. To a certain extent, the absorption characteristics reproduced the angular dependence of FEF. The transmittance presented in Figure 2c vanished at a greater angle than that of the TIR. The angular distribution of the horizontal ellipses had a similar shape to that of a resonance curve. For other cross-sections, we saw a substantial broadening. The curve was wider for the vertical ellipses, and in FEF and absorption.

## 3. Anisotropic Layer

The Helmholz equation gives dispersion relations in each part of the area, and boundary conditions match tangential components of electric and magnetic vectors E,H, respectively:

$$\begin{array}{ccc} -E_{0}\cos\theta +E_{R}\cos\theta & = & -\frac{D}{\varepsilon }\cos\psi -\frac{D_{R}}{\varepsilon }e^{\delta }\cos\psi , \end{array}$$

(2) $$\begin{array}{ccc} -\frac{D}{\varepsilon }e^{\delta }\cos\psi -\frac{D_{R}}{\varepsilon }\cos\psi & = & -E_{T}\cos\phi \;,\\ \varepsilon_{1}E_{0}\sin\theta +\varepsilon_{1}E_{R}\sin\theta & = & D\sin\psi -D_{R}e^{\delta }\sin\psi , \end{array}$$

(3) $$\begin{array}{ccc} De^{\delta }\sin\psi -D_{R}\sin\psi & = & \varepsilon_{2}E_{T}\sin\phi \;. \end{array}$$

We consider the scattering of incident wave with electric field $E_{0}$ by an isotropic or anisotropic layer of thickness *h*.

The model was a thin film with permittivity ε for the isotropic material. In the anisotropic case, the permittivity tensor had two diagonal components, $\varepsilon_{3}^{xx}=\varepsilon ,\varepsilon_{3}^{yy}=\varepsilon^{⊥}$. The incidence angle was θ, the inside layer refraction angle was ψ, and the transmittance angle was ϕ. Electric fields of reflected and transmitted waves are $E_{R}$ and $E_{T}$, respectively. Vectors of electric displacement D and $D_{R}$ were orthogonal to their wavevectors inside the layer.

**Figure 3:**
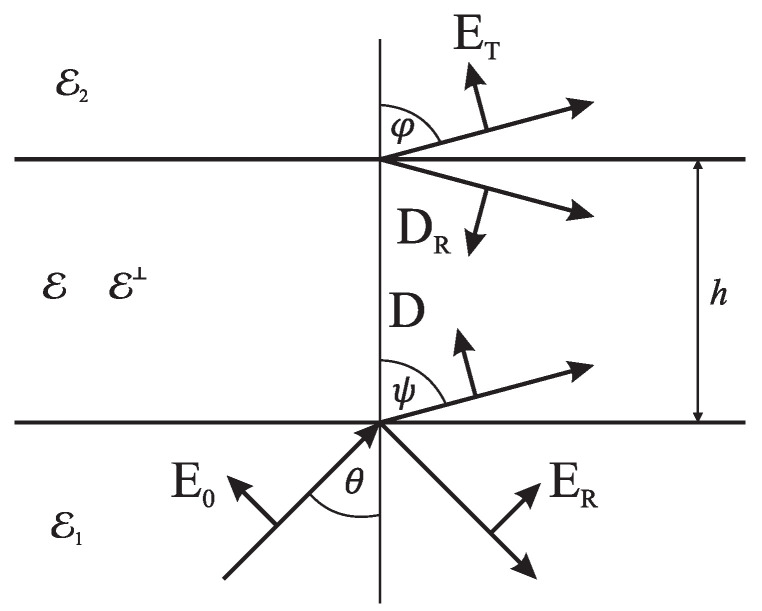
Anisotropic Fresnel’s model of a layer between two half-spaces. Permittivity was ε for parallel electric field and $\varepsilon^{⊥}$ for transverse field concerning the boundary.

Figure 3 illustrates the electric field notation: we calculated tangential components by multiplying vector lengths by cosines and sines of corresponding refraction angles. The imaginary part of the cosine or sine turns to zero only in nonabsorptive dielectric domains at refraction angles up to TIR. In general, they are complex and should be carefully defined. Furthermore, due to nonzero layer thickness, we take the phase shift into account:

(4) $$\delta =i\frac{2\pi }{\lambda }h\;\sqrt{\varepsilon -\varepsilon_{1}\frac{\varepsilon }{\varepsilon^{⊥}}\sin^{2}\theta }\;.$$

Complex angle ψ inside anisotropic layer satisfies relations

(5) $$\sin\psi =\frac{\sqrt{\varepsilon_{1}}\sin\theta }{\sqrt{\varepsilon +\varepsilon_{1}\left(1-\frac{\varepsilon }{\varepsilon^{⊥}}\right)\sin^{2}\theta }}\;,\;\cos\psi =\frac{\sqrt{\varepsilon -\varepsilon_{1}\frac{\varepsilon }{\varepsilon^{⊥}}\sin^{2}\theta }}{\sqrt{\varepsilon +\varepsilon_{1}\left(1-\frac{\varepsilon }{\varepsilon^{⊥}}\right)\sin^{2}\theta }}\;,$$

where the cut in the complex plane of the square root function goes along the negative real semiaxis. For the upper half-space, the transmit refraction angle is

(6) $$\sin\phi =\frac{\sqrt{\varepsilon_{1}}}{\sqrt{\varepsilon_{2}}}\;\sin\theta \;,\;\cos\phi =\frac{\sqrt{\varepsilon_{2}-\varepsilon_{1}\sin^{2}\theta }}{\sqrt{\varepsilon_{2}}}\;.$$

Equations (2) and (3) are sufficient to find transmittance

(7) T=4Re(ε2cosϕ)Re(ε1cosθ)×ε2ε1+cosϕcosθcoshδ−εε1sinψsinθcosϕcosψ+ε2εsinϕsinψcosψcosθsinhδ−2,

and absolute reflectance

(8) $$R={\left|\frac{\left(\frac{\sqrt{\varepsilon_{2}}}{\sqrt{\varepsilon_{1}}}-\frac{\cos\phi }{\cos\theta }\right)\cosh\delta -\left(\frac{\varepsilon }{\varepsilon_{1}}\frac{\sin\psi }{\sin\theta }\frac{\cos\phi }{\cos\psi }-\frac{\varepsilon_{2}}{\varepsilon }\frac{\sin\phi }{\sin\psi }\frac{\cos\psi }{\cos\theta }\right)\sinh\delta }{\left(\frac{\sqrt{\varepsilon_{2}}}{\sqrt{\varepsilon_{1}}}+\frac{\cos\phi }{\cos\theta }\right)\cosh\delta -\left(\frac{\varepsilon }{\varepsilon_{1}}\frac{\sin\psi }{\sin\theta }\frac{\cos\phi }{\cos\psi }+\frac{\varepsilon_{2}}{\varepsilon }\frac{\sin\phi }{\sin\psi }\frac{\cos\psi }{\cos\theta }\right)\sinh\delta }\right|}^{2}\;.$$

These coefficients mean the ratio of transmitted through the layer and reflected energy flux to the incident one. So, transmittance T, reflectance R, and absorption A give the unity. Then, to obtain absorption coefficient A=1−T−R. Here, absorption is specific, i.e., normalized by period *d* and cosθ. If phase shift δ tends to zero, Equations (7) and (8) become simpler and turn into Fresnel’s ones for two half-spaces with common boundary plane. If the anisotropic layer becomes isotropic $\varepsilon =\varepsilon^{⊥}$, then transmittance (7) coincides with the found one for magnetic field [20,21]. The model of the stratified medium with scalar dielectric constant is widely used in calculations of reflectometer sensitivity [22]. For reflectometry sensors based on a metallic–dielectric structure, ref. [23] the model agrees with measurements. Formulas for anisotropic dielectric permittivity tensors are used in papers devoted to hyperbolic metamaterials.

Let us plot the angular distribution within the isotropic and anisotropic layer models, and compare them with numerical results. We fit the model parameters (thickness and permittivity) to minimize the offsets by the least-squares method. The functional includes both the residues of transmission and specific absorption coefficients with equal weights:

(9) $$F=\sum_{n=1}^{N}{\left|T\left(\theta_{i}\right)-T_{i}\right|}^{2}+\sum_{n=1}^{N}{\left|A\left(\theta_{i}\right)-A_{i}\right|}^{2}\;.$$

where T(θ),A(θ) are the model transmittance or specific absorption depending on refraction angle, $T_{i},A_{i}$ are the transmittance or absorption calculated by BEM numerically at the incidence angle $\theta_{i}$ in the glass. Number N=201 corresponds to numerical samples of incidence angle in our calculations. We gathered the best-fit parameters of the least-squares procedure in Table 1.

**Figure 4:**
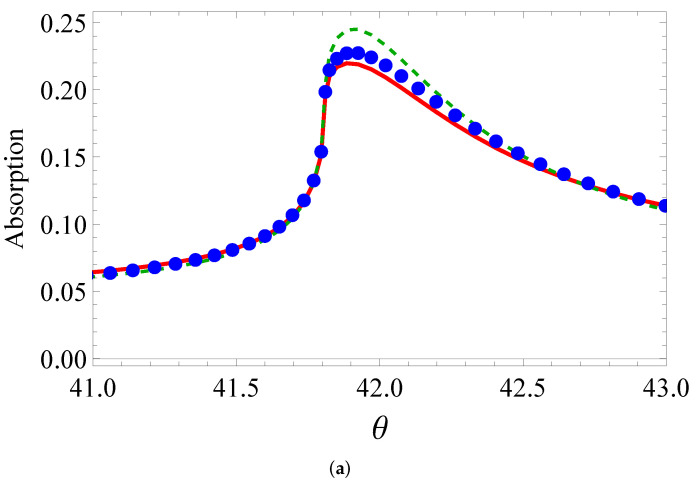

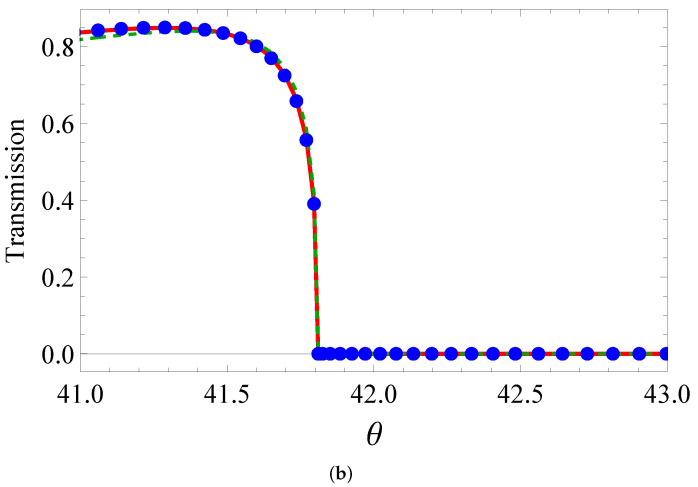
(**a**) Absorption and (**b**) transmission of circular cylinders as a function of the incidence angle θ (degrees): BEM calculation (solid line), isotropic layer (dashes), nonisotropic medium (circles).

**Figure 5:**
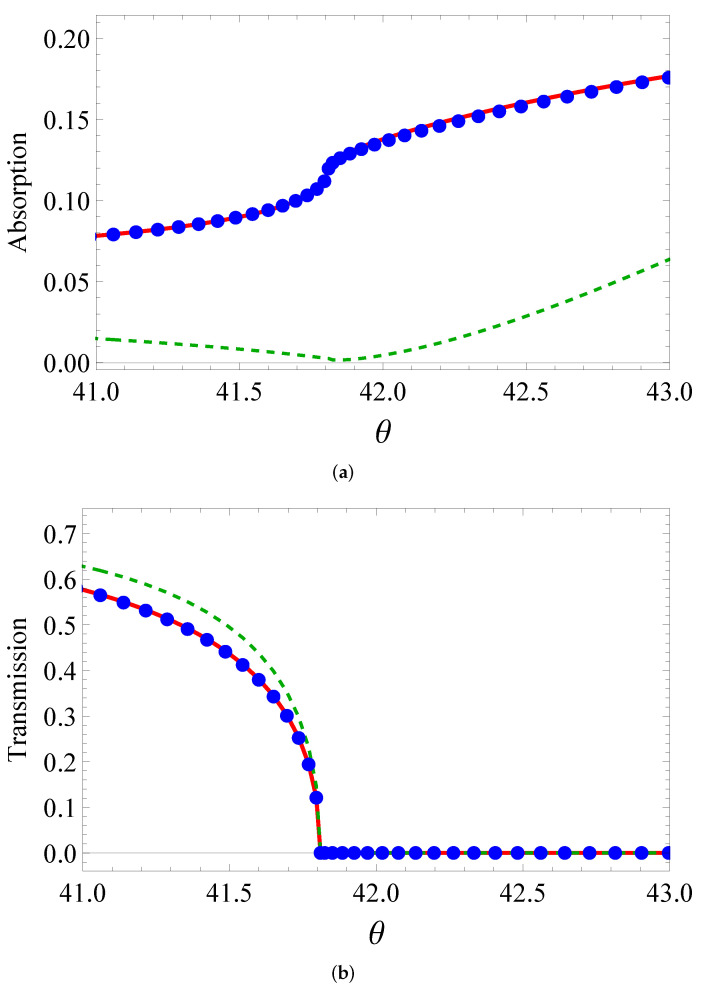
Same as in Figure 4, but for elliptic cylinders with vertical orientation of large axis.

**Figure 6:**
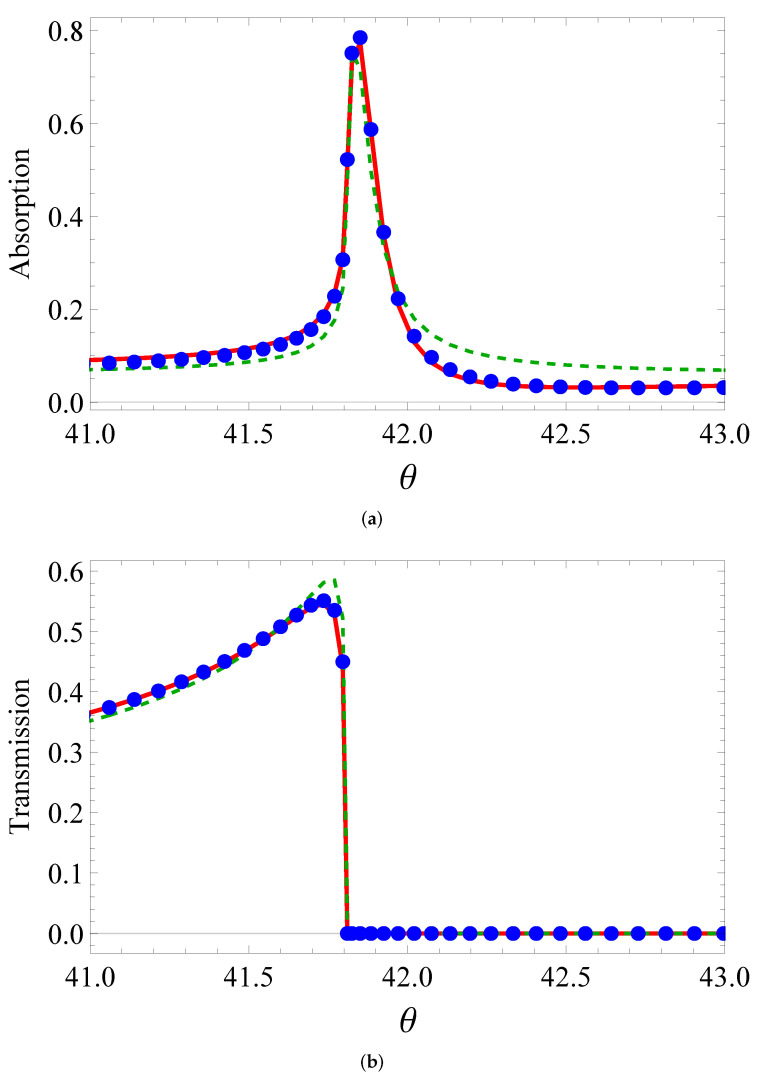
Same as in Figure 4, but for elliptic cylinders with horizontal orientation of large axis.

One can see how accurate the fit for absorption and transmission is from Figure 4, Figure 5 and Figure 6. The isotropic model qualitatively describes the BEM curves. When the real part of permittivity is negative (the third line and third column of the table), the fitting is satisfactory in transmission, but loses its accuracy in absorption. Otherwise, the curve comes to a huge mismatch. For example, if the real part becomes negative for vertical ellipses, the dashed curve comes in the bottom of Figure 5a. Meanwhile, the anisotropic model quantitatively describes all the curves with high accuracy. This is evident from the bottom line in Table 1. The better fit by the anisotropic model than that by the isotropic one was at least due to to more fitting parameters (5 vs. 3).

Anisotropy was sufficiently high in this structure. Different signs of the real part in the second and third lines from the bottom indicated that the media behaved as hyperbolic metamaterials, i.e., media with the hyperbolic dispersion law [24]. The circuit diagram could explain this observation within the concatenated capacitor model. For circular cylinders (nanowires) at a small fill-fraction of the metal compared to dielectric media in a unit cell, the Maxwell–Garnett approach is exploited in the calculation of dielectric tensor components for metamaterials [25]. In recent experiments on subwavelength imaging in the visible range [26], the anisotropic model was useful.

## 4. Conclusions

To study the angular dependence of scattering parameters near the TIR angle, we performed numerical calculations with the BEM. We considered circular and elliptic cylinders of a 4:1 axis ratio and treated changes in absorption, transmittance, and field enhancement factor. In addition, we analyzed two possible directions of the large elliptical axis: horizontal and vertical. Plasmonic resonance in transmission and absorption characteristics was sharper for horizontal ellipses, where the curvature radius at the slit was minimal.

We compared the calculated absorption and transmission coefficient with the “sandwich” model. Instead of the parallel wires, we placed a medium with some effective average permittivity and fixed thickness between two dielectric half-spaces and fit the data. This model allows for the analytical expressions of transmission and absorption coefficients. Least-squares minimisation showed that both models reproduced the behaviour of curves well. Furthermore, the fitted dependence for the anisotropic model practically coincided with BEM computation. Thus, the anisotropic model described the grating of parallel subwavelength wires.

## Figures and Tables

**Table 1:** Variational parameter results in isotropic and anisotropic layer models.

Parameter	Circle	Vertical Ellipse	Horizontal Ellipse
Isotropic/anisotropic layer thickness, nm (h)	55/40	9.2/103	24/19
Isotropic permittivity (ε)	20.7+0.7i	−27.6+1.5i	93.7+8.6i
Longitudinal permittivity (ε)	27.0+1.0i	−0.95+0.08i	117+19.1i
Transversal permittivity $\left(\varepsilon^{⊥}\right)$	−2.55+0.0i	1.34+0.04i	−2.96−0.55i
Isotropic/anisotropic layer distortion functional $\left(F\cdot 10^{3}\right)$	65.3/4.7	705/0.67	149/11.5

## Data Availability

Not applicable.

## References

[1] Zayats A.V., Smolyaninov I.I., Maradudin A.A. (2005). Nano-optics of surface plasmon polaritons. Phys. Rep..

[2] Fan X., Zheng W., Singh D.J. (2014). Light scattering and surface plasmons on small spherical particles. Light Sci. Appl..

[3] Chau Y.F., Yeh H.H. (2011). A comparative study of solid-silver and silver-shell nanodimers on surface plasmon resonances. J. Nanoparticle Res..

[4] Chou Chau Y.F., Chou Chao C.T., Huang H.J., Kooh M.R.R., Kumara N., Lim C.M., Chiang H.P. (2020). Perfect dual-band absorber based on plasmonic effect with the cross-hair/nanorod combination. Nanomaterials.

[5] Gupta B.D., Kant R. (2018). Recent advances in surface plasmon resonance based fiber optic chemical and biosensors utilizing bulk and nanostructures. Opt. Laser Technol..

[6] Xu Y., Bai P., Zhou X., Akimov Y., Png C.E., Ang L.K., Knoll W., Wu L. (2019). Optical refractive index sensors with plasmonic and photonic structures: Promising and inconvenient truth. Adv. Opt. Mater..

[7] Zhao Y., Tong R.j., Xia F., Peng Y. (2019). Current status of optical fiber biosensor based on surface plasmon resonance. Biosens. Bioelectron..

[8] Peralta E.A., Soong K., England R.J., Colby E.R., Wu Z., Montazeri B., McGuinness C., McNeur J., Leedle K.J., Walz D. (2013). Demonstration of electron acceleration in a laser-driven dielectric microstructure. Nature.

[9] Rovey J.L., Friz P.D., Hu C., Glascock M.S., Yang X. (2015). Plasmonic force space propulsion. J. Spacecr. Rocket..

[10] Prodan E., Radloff C., Halas N.J., Nordlander P. (2003). A hybridization model for the plasmon response of complex nanostructures. Science.

[11] Chau Y.F., Jheng C.Y., Joe S.F., Wang S.F., Yang W., Jheng S.C., Sun Y.S., Chu Y., Wei J.H. (2013). Structurally and materially sensitive hybrid surface plasmon modes in periodic silver-shell nanopearl and its dimer arrays. J. Nanoparticle Res..

[12] Ho Y.Z., Chen W.T., Huang Y.W., Wu P.C., Tseng M.L., Wang Y.T., Chau Y.F., Tsai D.P. (2012). Tunable plasmonic resonance arising from broken-symmetric silver nanobeads with dielectric cores. J. Opt..

[13] Bogaerts W., Chrostowski L. (2018). Silicon photonics circuit design: Methods, tools and challenges. Laser Photonics Rev..

[14] Frumin L., Shapiro D. (2020). Sensitivity enhancement of plasmonic grating in the local field. Opt. Express.

[15] Frumin L., Nemykin A., Perminov S., Shapiro D. (2013). Plasmons excited by an evanescent wave in a periodic array of nanowires. J. Opt..

[16] Nemykin A., Perminov S., Frumin L., Shapiro D. (2015). Excitation of a plasmon resonance in metal cylinders by an evanescent wave. Quantum Electron..

[17] Palik E.D. (1998). Handbook of Optical Constants of Solids.

[18] Bohren C.F., Huffman D.R. (2004). Absorption and Scattering of Light by Small Particles.

[19] Zymovetz S.V., Geshev P.I. (2006). Boundary Integral Equation Method for Analysis of Light Scattering by 2D Nanoparticles. Tech. Phys..

[20] Kotkin G.L. (1966). On excitation of surface wave. Phys. Met. Metallogr..

[21] Raether H. (1988). Surface Plasmons on Smooth and Rough Surfaces and on Gratings.

[22] Shalabney A., Abdulhalim I. (2010). Electromagnetic fields distribution in multilayer thin film structures and the origin of sensitivity enhancement in surface plasmon resonance sensors. Sens. Actuators A Phys..

[23] Terentyev V.S., Simonov V.A. (2021). Spectral Characteristics of an Oblique-Incidence Reflection Interferometer as a Refractive Index Sensor. Opt. Spectrosc..

[24] Poddubny A., Iorsh I., Belov P., Kivshar Y. (2013). Hyperbolic metamaterials. Nat. Photonics.

[25] Cortes C.L., Newman W., Molesky S., Jacob Z. (2012). Quantum nanophotonics using hyperbolic metamaterials. J. Opt..

[26] Bronnikov K., Arriaga J., Krokhin A., Drachev V.P. (2021). Sub-Diffraction-Limit Imaging System with two Interfacing Hyperbolic Metamaterials. Phys. Rev. Appl..

